# Systematic review and meta-analysis of childhood exposure to antibiotics and the subsequent risk of IBD

**DOI:** 10.1093/ibd/izaf324

**Published:** 2026-01-31

**Authors:** Ketil Størdal, Svend Andersen, Karl Mårild, Vilhelm Larsson, Henrik Imberg

**Affiliations:** Department of Pediatric Research, Faculty of Medicine, University of Oslo, Oslo, Norway; Children’s Center, Oslo University Hospital, Oslo, Norway; Department of Pediatrics, Vestfold Hospital Trust, Tønsberg, Norway; Institute of Clinical Medicine, University of Oslo, Oslo, Norway; Department of Pediatrics, Institute of Clinical Sciences, Sahlgrenska Academy, University of Gothenburg, Gothenburg, Sweden; Department of Pediatrics, Queen Silvia Children’s Hospital, Gothenburg, Sweden; Statistiska Konsultgruppen Sweden AB, Gothenburg, Sweden; Statistiska Konsultgruppen Sweden AB, Gothenburg, Sweden; Department of Molecular and Clinical Medicine, Institute of Medicine, Sahlgrenska Academy, University of Gothenburg, Gothenburg, Sweden

**Keywords:** antibiotic, child, inflammatory bowel disease, Crohn’s disease, ulcerative colitis

## Abstract

**Background:**

Antibiotic use in early childhood may alter the developing microbiome and has been proposed as a risk factor for inflammatory bowel disease (IBD). We conducted a systematic review to examine the association between childhood antibiotic use and subsequent risk of IBD.

**Methods:**

In a systematic literature search, we identified cohort and case-control studies reporting the association between antibiotic use (exposure age <1 to 17 years) and development of IBD. MEDLINE and EMBASE databases were searched from inception through December 31, 2024. Studies reporting a hazard ratio, odds ratio, or risk ratio (RR) were included. To account for heterogeneity, pooled estimates were calculated using the DerSimonian-Laird random-effects model. Estimates were adjusted for potential confounding as reported in the original studies.

**Results:**

We identified 10 studies, of which 8 (n = 2783 cases) reported associations between childhood antibiotics and IBD risk. Additionally, 2 studies on Crohn’s disease (CD) and 1 on ulcerative colitis were included in disease-specific analyses. In pooled analyses, antibiotic exposure compared with no exposure was associated with increased risk of IBD (RR, 1.42; 95% confidence interval [CI], 1.23-1.66), CD (RR, 1.59; 95% CI, 1.39-1.81), and ulcerative colitis (RR, 1.23; 95% CI, 1.08-1.40). Heterogeneity was low to moderate (I^2^ = 0%-35%), and funnel plots did not indicate publication bias (Egger’s test, *P = *.12-.43). Adjustment for infections did not attenuate the association between childhood antibiotic exposure and IBD development.

**Conclusions:**

While causal interpretation should be cautious, childhood exposure to antibiotics was associated with an increased risk of later IBD, particularly for CD.

Key Messages **What is already known?**

The risk of inflammatory bowel disease (IBD) is modified by the environment, and growing evidence supports that an abnormal recognition of commensal microbes may play a role.

**What is new here?**

The overall evidence suggests that early childhood antibiotic exposure is associated with increased risk of IBD, particularly Crohn’s disease. Studies suggest a stronger association with early compared with later childhood antibiotics, and with repeated compared with single antibiotic exposures.

**How can this study help patient care?**

Cautious use of antibiotics may be important to avoid potential long-term risks such as IBD, underscoring the importance of judicious antibiotics use.

## Introduction

Inflammatory bowel disease (IBD), which includes Crohn’s disease (CD), ulcerative colitis (UC), and IBD unclassified, is a chronic condition characterized by intestinal inflammation and extraintestinal manifestations, often presenting in young adulthood.^1^ The incidence of childhood-onset IBD has been increasing, whereas in recent years, the incidence of adult-onset IBD appears to have plateaued in high-income countries.^2^^,^^3^

Though genetic susceptibility is important, the increasing prevalence cannot be attributed to identified genetic factors, prompting investigations into environmental contributors and gene-environment interactions.^4^ Functional genetic studies have showed that impaired recognition of commensal microbes may be an important pathogenic mechanism of IBD.^5^^,^^6^ As a result, alterations in the composition of the microbiome, particularly during an early window of susceptibility, are considered central to the pathophysiology of IBD.^6^

During the first years of life, the gut microbiome undergoes rapid maturation toward an adult-like composition and is shaped by the child’s living environment.^7^ Key determinants influencing the microbiome development include mode of delivery, breastfeeding, and the composition of the weaning diet.^8^ Notably, antibiotic exposure during infancy and early childhood exerts more pronounced and sustained effects on the microbiome compared with exposures later in life.^9^^,^^10^ Consequently, early life represents a critical window of vulnerability during which environmental factors can modulate the developing gut microbiome.

Several studies have examined the composition of the gut microbiome at the time of IBD diagnosis, consistently demonstrating a microbial signature different from healthy individuals.^11^ This evidence is insufficient to establish specific microbial patterns as causal, as dysbiosis may be a consequence rather than a cause of intestinal inflammation. However, emerging data from studies investigating the microbiome in predisease states of IBD suggest causal relationships.^11^ Increased susceptibility to infections observed in some individuals who go on to develop IBD complicates the interpretation of associations between antibiotic use and subsequent IBD risk.

The aim of this systematic review and meta-analysis was to examine the association between childhood antibiotic exposure and the subsequent risk of developing IBD. In addition, we aimed to investigate potential differences in this association between CD and UC. Finally, we sought to explore whether the observed association might be confounded by an underlying predisposition to infections. Several large cohort studies published the last 2 years have not been included in previous systematic reviews, calling for updated analyses.

## Methods

**Figure 1.**
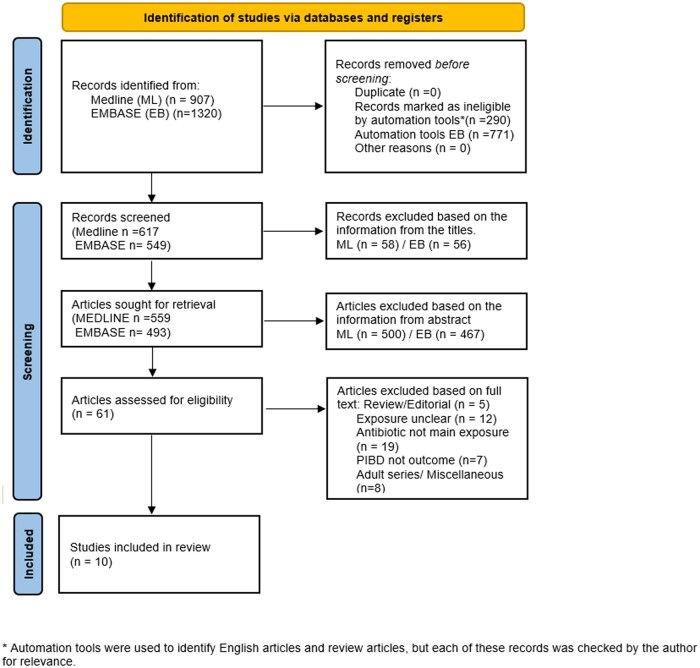
PRISMA 2020 flow diagram illustrating the selection process of studies included in the meta-analysis. PIBD, pediatric IBD.

A systematic literature search was performed by 2 independent researchers (S.A. and K.S.) using MEDLINE and EMBASE, covering the period from database inception to December 31, 2024. Study selection followed the PRISMA 2020 statement,^12^ as illustrated in Figure 1. In addition to the database search, we included articles identified through manual screening of reference lists in previous publications, including prior systematic reviews and meta-analyses. No specific preregistration or study protocol were registered.

### Eligibility criteria

We included observational studies (cohort or case-control) that reported the association between antibiotic use in childhood (age of exposure <1 to 2 years in most studies) and development of IBD before 18 years of age. Studies conducted in adult populations were included only if they presented separate subanalyses for children and adolescents. Studies reporting exclusively on CD or UC were excluded from the main meta-analysis but included in disease-specific analyses. Studies were eligible if they reported adjusted effect estimates as odds ratios, risk ratios (RRs), or hazard ratios (HRs). We allowed inclusion of studies even if cohorts could be partially overlapping with other studies.

### Data extraction

Two reviewers (K.S. and S.A.) independently completed data extraction using a standardized data collection form. The extracted data included the following^1^: study characteristics, such as the primary author, year of publication, study design (cohort or case-control), study population (adult, pediatric, or both), and the study location^2^; sample sizes, including the total population^3^; source of exposure data^4^; IBD subtype and age group^5^; adjustment variables^6^; and risk estimates with their corresponding confidence intervals (CIs).

We examined the included studies for data on the association between specific classes of antibiotics and the risk of IBD. Where available, we extracted information regarding the associations for single compared with repeated antibiotic exposures. Given that antibiotics are typically prescribed in the context of a suspected bacterial infection, infections may confound the observed association between antibiotic use and IBD. Therefore, we specifically assessed whether the studies accounted for the frequency of infections or reported the indications for antibiotic prescription.

### Quality assessment

Two authors (K.S. and S.A.) independently assessed the methodological quality of the included studies using the Newcastle-Ottawa Scale for cohort and case-control studies.^13^ The scale consists of 8 items evaluating 3 domains: selection (4 items, maximum score of 4), comparability (1 item, maximum score of 2), and outcome/exposure (3 items, maximum score of 3). Studies were graded as good quality if they received 3 or 4 stars in the selection domain, 1 or 2 stars in the ­comparability domain, and 2 or 3 stars in the outcome/­exposure domain.

### Statistical analyses

Pooled effect estimates were calculated using a random-effects model with the DerSimonian-Laird estimator to account for between-study heterogeneity. Given the low absolute incidence of IBD in the general population, HRs and odds ratios were considered to approximate RRs. Estimates were synthesized on the logarithmic scale and exponentiated to obtain a pooled RR. When multiple estimates were reported within a study, those derived from adjusted models were prioritized. Standard errors were calculated from the reported confidence intervals when not directly provided. Sensitivity analyses of model choice were performed using restricted maximum likelihood estimation and the Hartung-Knapp adjustment for standard errors and confidence intervals. Between-study heterogeneity was assessed using the I^2^ statistic, which quantifies the proportion of total variation attributable to heterogeneity rather than chance and was tested using Cochran’s Q test. According to conventional thresholds, I^2^ values of 25%, 50%, and 75% were interpreted as indicating low, moderate, and substantial heterogeneity, respectively.^14^ The influence of individual studies on the overall effect and heterogeneity was evaluated using a leave-one-out sensitivity analysis, whereby each study was sequentially excluded and the pooled estimate and I^2^ recalculated.

Potential publication bias was examined using funnel plots, in which log-transformed effect estimates were plotted against their standard errors. Visual asymmetry was evaluated in conjunction with Egger’s regression test to detect small-study effects.

To evaluate the potential impact of unmeasured confounding, we calculated the E-value for each pooled estimate. The E-value quantifies the minimum strength of association, on the RR scale, that an unmeasured binary confounder would need to have with both the exposure and the outcome—conditional on the measured covariates—to fully account for the observed association.

All analyses were conducted in R version 4.4.1 (R Foundation for Statistical Computing), with meta-analyses implemented using the metafor package version 4.8.0.

## Results

Out of a total of 465 studies screened, 8 (n = 2783 cases) met the inclusion criteria for the meta-analysis examining childhood antibiotic exposure and the risk of IBD, as illustrated in the study selection flowchart (Figure 1). Additionally, 2 studies focusing exclusively on CD and 1 on UC were included in disease-specific analyses alongside the 8 broader studies. All included studies met the Newcastle-Ottawa Scale criteria and were deemed to be of good quality (Table S1). The main limitation was inadequate information on nonresponse rates in case-control studies, and unclarities in case/control definition in 2 of these studies.

The defined age of antibiotic exposure was under 2 years in 6 studies^15-20^ and up to 17 years in the others.^21-24^ Six studies originated from the Nordic countries, characterized by a restrictive use of antibiotics. Detailed characteristics of the included studied are provided in Table S2.^15-24^

### Antibiotics and risk of IBD

**Figure 2.**
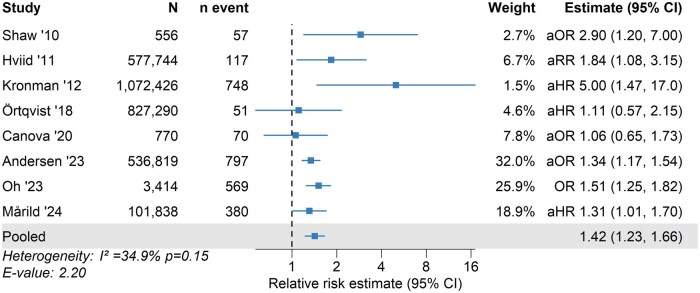
Association between childhood antibiotic exposure and risk of inflammatory bowel disease. Squares and horizontal lines represent study-specific relative risk estimates (reported as hazard ratio, odds ratio [OR], or risk ratio) with 95% confidence intervals (CIs), with the pooled risk ratio shown at the bottom. Studies are listed in chronological order by publication year. aHR, adjusted hazard ratio; aOR, adjusted odds ratio; aRR, adjusted risk ratio.

**Figure 3.**
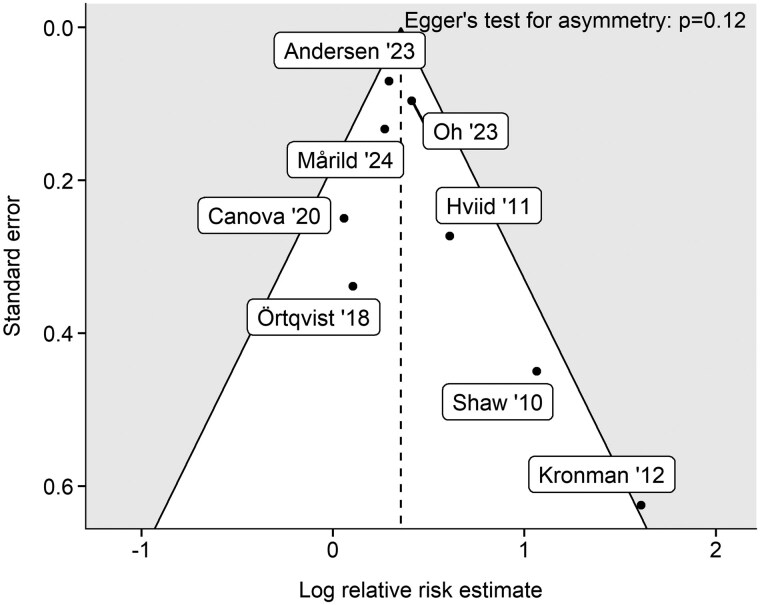
Funnel plot of childhood antibiotic exposure and risk of inflammatory bowel disease. Each point represents a study-specific relative risk estimate (log hazard ratio, log odds ratio, or log risk ratio) plotted against its standard error. The dashed vertical line shows the pooled log risk ratio. Diagonal lines depict the expected 95% range assuming no heterogeneity. Points falling within the gray area may indicate outliers, small-study effects, or between-study heterogeneity.

Across the 8 studies included in the main analysis, 6 reported statistically significant positive associations between childhood antibiotic exposure and subsequent IBD risk. The pooled RR for IBD comparing antibiotic exposure to no exposure was 1.42 (95% CI, 1.23-1.66) (Figure 2). Between-study heterogeneity was moderate (I^2^ = 35%; *P = *0.15), and the funnel plot showed no significant asymmetry (Egger’s test, *P = *0.12) (Figure 3). The E-value for the observed association was 2.20, indicating that an unmeasured binary confounder would need to be associated with both antibiotic exposure and IBD by a RR of at least 2.20 to fully explain the observed association.

Sensitivity analyses identified 1 study^21^ as a notable outlier, reporting a large effect estimate (adjusted HR [aHR], 5.00) with a wide CI (1.47-17.0), despite its substantial sample size (n = 1 072 426), and falling outside the expected 95% range in the funnel plot (Figure 3). However, the study primarily contributed to heterogeneity, which dropped to 8.6% after its exclusion, while the pooled RR remained virtually unchanged (adjusted RR, 1.39; 95% CI, 1.25-1.55) (Figure S1).

Among the studies focusing on antibiotic exposure before the age of 2 years,^15-19^ 4 out of 5 studies with IBD as outcome reported a significant positive association with subsequent IBD risk. Of note, 2 studies indicated a more pronounced association of antibiotic exposure during the first year of life with later IBD compared with antibiotics later in life.^18^^,^^21^

### Crohn’s disease

**Figure 4.**
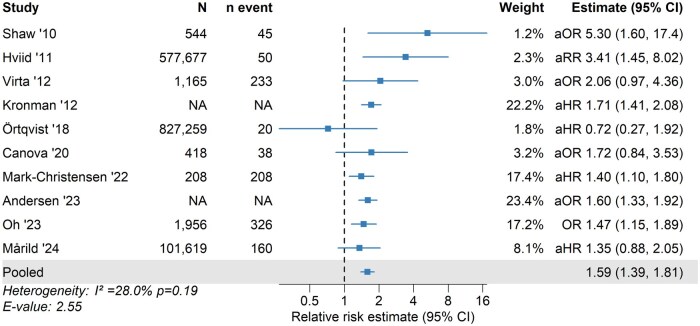
Association between childhood antibiotic exposure and risk of Crohn’s disease. Squares and horizontal lines represent study-specific relative risk estimates (reported as hazard ratio, odds ratio [OR], or risk ratio [RR]) with 95% confidence intervals (CIs), with the pooled RR shown at the bottom. Studies are listed in chronological order by publication year. aHR, adjusted hazard ratio; aOR, adjusted odds ratio; aRR, adjusted risk ratio; NA, Not Available.

For CD, 6 out of the 10 included studies reported statistically significant positive associations with childhood antibiotic exposure. The pooled analysis demonstrated a 1.59-fold increased risk (95% CI, 1.39-1.81) associated with antibiotic exposure (Figure 4). Between-study heterogeneity was moderate (I^2^ = 28%; *P = *0.19), and the funnel plot (Figure S2) showed no evidence of asymmetry (Egger’s test, *P = *0.26). One study^15^ was a potential outlier, but the leave-one-out sensitivity analyses showed limited influence of individual studies on the pooled estimate or heterogeneity (Figure S3). The E-value was 2.55, suggesting that unmeasured confounding would need to be associated with both antibiotic exposure and CD by an RR of at least 2.55 to fully explain the observed association.

Among studies restricted to antibiotic exposure before 2 years of age,^13-17^ 4 of 6 reported a significant positive association with subsequent risk of CD.

### Ulcerative colitis

**Figure 5.**
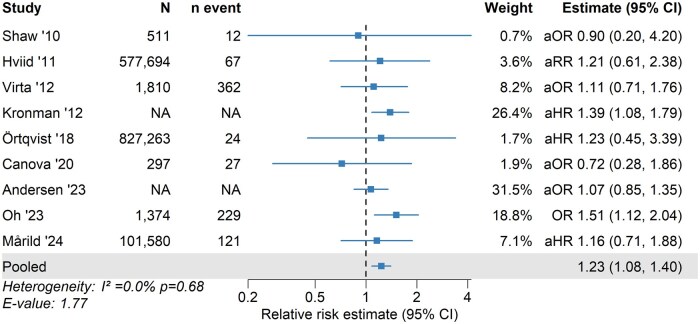
Association between childhood antibiotic exposure and risk of ulcerative colitis. Squares and horizontal lines represent study-specific relative risk estimates (reported as hazard ratio, odds ratio [OR], or risk ratio) with 95% confidence intervals (CIs), with the pooled risk ratio shown at the bottom. Studies are listed in chronological order by publication year. aHR, adjusted hazard ratio; aOR, adjusted odds ratio; aRR, adjusted risk ratio; NA, Not Available.

For UC, statistically significant positive associations with antibiotic exposure were observed in 2 out of 9 included studies. Overall, effect estimates for UC showed greater variability compared with those observed for CD. In the pooled analysis, antibiotic exposure was associated with a 1.23-fold increased risk of UC (95% CI, 1.08-1.40) (Figure 5). No statistical heterogeneity was detected (I^2^ = 0.0%; *P = *0.43), and the funnel plot demonstrated a symmetric distribution (Egger’s test, *P = *0.66) (Figure S4). Leave-one-out sensitivity analyses (Figure S5) showed consistent results upon sequential exclusion of individual studies. The E-value was 1.77, suggesting that unmeasured confounding would need to be associated with both antibiotic exposure and UC by a RR of at least 1.77 to fully account for the observed association.

### Type of antibiotics and dose-response associations

Several studies suggested that the association between early life antibiotic exposure and subsequent IBD risk may vary by antibiotic class. However, differences in antibiotic classification across studies precluded a meta-analysis of specific drug classes (Table S1). Penicillins and extended-spectrum penicillins were the most frequently prescribed antibiotics. Three studies reported weaker associations for these narrow-spectrum antibiotics compared with broader-spectrum agents,^17^^,^^19^^,^^24^ while 1 study found an increased risk associated with penicillin use.^18^ For macrolides, the reported associations varied: weaker,^21^^,^^22^ stronger,^20^ or comparable to other antibiotic classes.^17^^,^^19^^,^^23^

A dose-response association was observed; all 6 studies that found an overall positive association with early-life antibiotics and IBD reported an increased risk associated with repeated antibiotics (≥2 courses), compared with single courses.^15^^,^^17-19^^,^^21^^,^^22^ The study by Canova et al.^6^ which found no overall association of any antibiotic use with IBD, observed a significantly increased risk among individuals exposed to 4 or more antibiotic courses compared with no use.

Sensitivity analyses using restricted maximum likelihood estimation and/or the Hartung-Knapp adjustment to the standard errors and CIs for pooling in the random-effects model produced virtually identical results (Figure S6).

### Infections and confounding by indication

Given that antibiotics are typically prescribed in the context of suspected bacterial infections, infections may act as a confounder in the observed association. Only 1 study addressed this directly by linking reported infection history from early childhood to prospectively collected IBD outcome data.^18^ The frequency of parent-reported infections during the first year of life (aHR, 1.01; 95% CI, 0.96-1.07) and between 1 and 3 years of age (aHR, 1.00; 95% CI, 0.99-1.01) was not associated with subsequent IBD risk. Adjustment for the frequency of infections had minimal impact on the association between antibiotic exposure and later IBD risk in analyses with (aHR, 1.33; 95% CI, 1.01-1.76) and without (aHR, 1.31; 95% CI, 1.01-1.70) adjustment for infection frequency.

## Discussion

In this systematic review and meta-analysis, we confirmed a positive association between childhood antibiotic exposure and an increased risk of developing IBD, as previously observed in several individual studies. The association was more pronounced for CD compared with UC, although significant associations were observed for both subtypes. The stronger association observed for CD compared with UC, in line with previous meta-analyses in adults, may suggest that microbiome influences are more important for CD. This also harmonizes with current insight in the function of major genetic variants in CD.^25^ The effect sizes for IBD and CD were generally consistent across studies, with no evidence of substantial heterogeneity or disproportionate influence from any single study on the overall estimates.

With the inclusion of several recently published studies from the past 2 years,^17-20^ this meta-analysis provides a more comprehensive and up-to-date evidence base than previous analyses.^26^ Unlike an earlier analysis that pooled unadjusted estimates,^27^ the present analysis incorporated adjusted effect estimates, which likely accounts for some of the discrepancies observed across individual studies. Adjustment for key confounders, such as birth year and family history of IBD, enhances the internal validity of individual study findings and contribute to more reliable pooled estimates. Furthermore, we assessed heterogeneity and evaluated the influence of individual studies, adding further confidence in the consistency and robustness of the findings.

This meta-analysis has several additional strengths, most notably the increased statistical power achieved by including nearly 2800 cases of childhood-onset IBD, even though half of the individual studies included relatively small sample sizes (50-117 cases). Although the exposure windows varied across studies, limiting direct comparability, a more consistent pattern emerged among the 5 studies assessing antibiotic exposure before 2 years of age: only 1 study with the smallest sample size did not demonstrate a significant association with IBD.^16^ Investigating this early age period of antibiotic use is important for several reasons. First, analyses restricted to exposure by 2 years of age are less susceptible to reverse causation (ie, antibiotic use prompted by subclinical symptoms of undiagnosed IBD). IBD onset before 2 years of age (infantile-onset IBD) is exceptionally rare, though the possibility of reverse causation cannot be completely excluded.^2^ Moreover, antibiotic exposure during this critical period may exert a stronger influence on the gut microbiome and the developing immune system than exposures occurring later in life. Importantly, all included studies reported a dose-response relationship between antibiotic exposure and IBD risk.

As with all meta-analyses, heterogeneity in study designs represents an important limitation, particularly regarding differences in the covariates used for adjustment. While some of the studies reported unadjusted estimates or adjusted solely for basic variables such as birth year and calendar period,^17^^,^^22^ the majority accounted for a broader range of potential confounders. Studies with a limited selection of confounders may therefore have produced inflated effect estimates. The extent of unmeasured confounding in our analysis appears to be small to moderate, as indicated by the E-values, which quantify the minimum strength of association that an unmeasured confounder would need to have with both the exposure and the outcome to fully explain the observed association. The E-values ranged from 1.77 to 2.55 across outcomes, suggesting that only confounders with relatively strong associations with both exposure and outcome could fully account for the observed effects. The inclusion of confounding assessed by the E-values is a notable strength compared with previous meta-analyses.^26-28^ We did not detect any signs of publication bias in funnel plots; however, the low number of included studies limits the sensitivity for such potential bias.

A notable source of outcome heterogeneity is the variation in upper age limits for case inclusion. For example, 1 study restricted its analysis to very early-onset IBD (< 6 years of age) and found an association with prenatal, but not postnatal, antibiotic exposure.^23^ In this subset of very early-onset IBD, monogenic disease is more common and may be less susceptible to environmental influences.^29^ We included only studies on IBD with onset <18 years age; previous data indicate a stronger association for childhood-onset IBD compared with adult-onset disease.^17^^,^^28^

The main limitation of this meta-analysis is the lack of information on the indications for antibiotic prescriptions. If children who later develop IBD are inherently more susceptible to bacterial infections, this could lead to confounding by indication. Rare immunodeficiency disorders, such as chronic granulomatous disorder or IL-10 receptor deficiency, are characterized by recurrent and severe bacterial infections and IBD.^30^ However, given the very low prevalence of these conditions, they are unlikely to account for the overall association observed in this analysis. Adjustment for childhood infections did not attenuate the association between childhood antibiotics and subsequent risk of IBD. However, this should be interpreted cautiously because only a single prospective cohort study included this confounder.^18^ Notably, hospitalization for infection before 3 years of age was independently associated with an increased risk of subsequent IBD, suggesting that this relationship warrants further investigation and independent replication.^18^ Furthermore, different phenotypes of CD and UC have not been detailed in the included studies, which in the future could increase our understanding of pathophysiology. Finally, preregistration of the study protocol would be desirable in accordance with recommendations.

The use of antibiotics during childhood remains high, with nearly a 10-fold difference between countries and regions, suggesting substantial unwarranted variation in use.^31^ In addition to the emerging risk of antimicrobial resistance, individual-level risks—such as the possible development of specific immune-mediated diseases—underscore the need for cautious antibiotic prescription.^27^^,^^32^ In several of the studies included in the current meta-analysis, antibiotic exposure during the first year of life was more strongly associated with subsequent IBD than exposures later in childhood.

Therefore, careful use of antibiotics during this critical developmental window, when the gut microbiome is most vulnerable to disruption, remains the key actionable takeaway. Caution remains necessary in the interpretation; residual confounding may distort the results of any study in which practical or ethical considerations prevent randomized exposure allocation. Furthermore, the associations between antibiotic exposure and IBD may be different in populations more exposed to antibiotics than in the areas represented in the current review, predominated by the Nordic countries.

Nonetheless, given the essential role of antibiotics in treating severe infections, strategies to mitigate potential harmful effects are also important.^33^ Interventions such as prebiotics, probiotics, synbiotics, or fecal transplants can help restore disruptions in the gut microbiome.^34^ The composition of the microbiome is also modified by mode of delivery, diet and external living environment.^33^ Whether susceptibility to the adverse effects of antibiotics on long-term health outcomes can be modified by diet and other exposomic factors remains an open question and important area for future research.

In conclusion, the meta-analysis found that childhood exposure to antibiotics is associated with an increased risk of developing IBD, with the strongest association observed for CD. Although causal interpretation should be made cautiously, these findings underscore the importance of judicious antibiotics use, particularly during the first years of life when the developing gut microbiome is most vulnerable.

## Supplementary Material

izaf324_Supplementary_Data
