# Supplementary material for: Systematic review and meta-analysis of childhood exposure to antibiotics and the subsequent risk of IBD
Source: Inflamm Bowel Dis. 2026 Jan 31;32(5):990–7. doi: 10.1093/ibd/izaf324 (PMC13135832; doi:10.1093/ibd/izaf324)
Supplement: izaf324_Supplementary_Data [file izaf324_supplementary_data.pdf]

**Table S1.** Studies included in the meta-analysis of early antibiotic exposure and later risk of inflammatory bowel disease, Crohn's disease and ulcerative colitis.

| Authors, year, setting, reference | Number of cases with IBD, CD or UC | Non-IBD controls | Age of antibiotic exposure | Age range of IBD outcome | Specific antibiotic types with stronger association |
|---|---|---|---|---|---|
| Shaw et al 2010, Canada(1) | IBD: n=36<br>CD: n=27<br>UC: n=9 | n=360 | <1 year. | 2-12 years.<br>Median 8.4 years. | |
| Hviid et al 2011, Denmark(2) | IBD: n=117<br>CD: n=50<br>UC: n=67 | n=577,510 | 0-9 years. | 0-9.8 years.<br>Mean 3.4 years. | Penicillin V and extended spectrum penicillins, not macrolides. |
| Kronman et al 2012, UK(3) | IBD: n=748<br>CD: n=449<br>UC: n=272 | n=1,070,930 | 0-≤17 years. | 0-≤17 years. | Stronger association for anti-anaerobics, and for exposure <1 year age. Latency period excluded. |
| Örtqvist et al 2018, Sweden(4) | IBD: n=51<br>CD: n=20<br>UC: n=24 | n=827,188 | <6 years. | <6 years (very early onset IBD) | No difference between penicillin V and any systemic antibiotic. |
| Canova et al 2020, Italy(5) | IBD: n=70<br>CD: n=38<br>UC: n=27 | n=700 | 0-6 months and 0-12 months. | 1-≤17 years.<br>Mean 8.8 years. | Increased risk by ≥4 courses and for CD. |
| Andersen et al 2023, Norway(6) | IBD: n=797<br>CD: n=464<br>UC: n=292 | n=536,022 | <2 years. | 2-≤16 years.<br>Median 11.4 years. | For all classes except sulphonamides and trimethoprim, strongest for "other broad-spectrum antibiotics" |
| Oh et al 2023, South Korea(7) | IBD: n=569<br>CD: n=326<br>UC: n=229 | n=2,845 | <1 year. | 0-≤14 years. | Significant only for broad-spectrum antibiotics. Macrolides estimates of same size as overall estimates. |
| Mårild et al 2024, Norway/Sweden(8) | IBD: n=395<br>CD: n=165<br>UC: n=129 | n=102,651 | <1 year. | 1-23 years. | Penicillins <1 year significant, but not for non-penicillins. |
| Virta et al 2012, Finland(9) | CD: n=233<br>UC: n=362 | n=2,380 | 0-≤16.5 years. | 0-≤16.5 years. | Strongest association for cephalosporins. |
| Mark-Christensen et al 2022, Denmark(10) | CD: n=208 | n=978,831 | <1 year. | 0-≤17 years. | Strongest association for macrolides, lincosamides and streptogramins combined. |

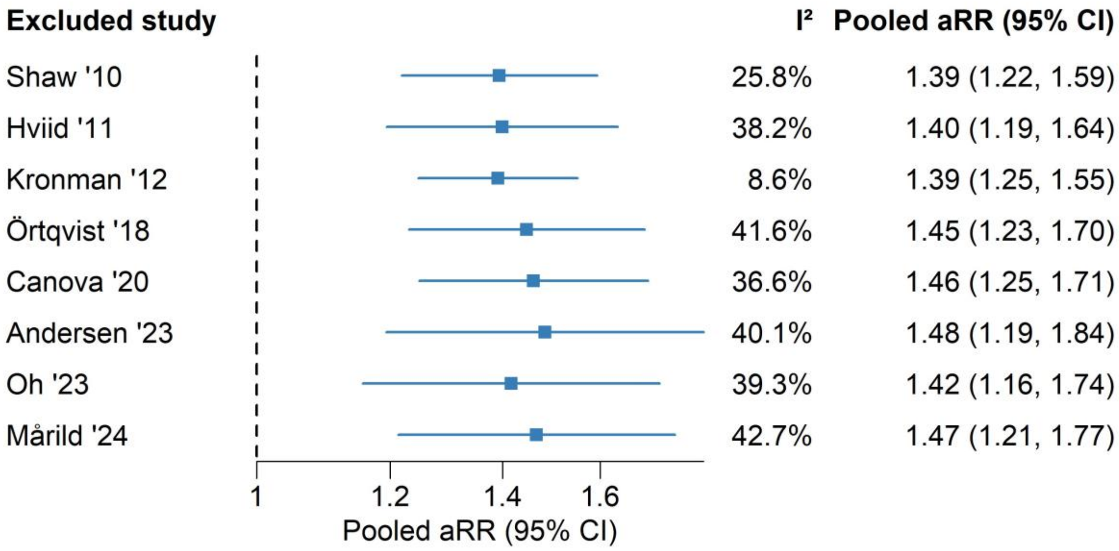


**Figure S1.** Leave-one-out sensitivity analysis of childhood antibiotic exposure and risk of inflammatory bowel disease. Squares and horizontal lines represent the pooled adjusted risk ratio (aRR) and 95% confidence interval (CI) after excluding the indicated study. $I^2$ values represent between-study heterogeneity after exclusion of the respective study.

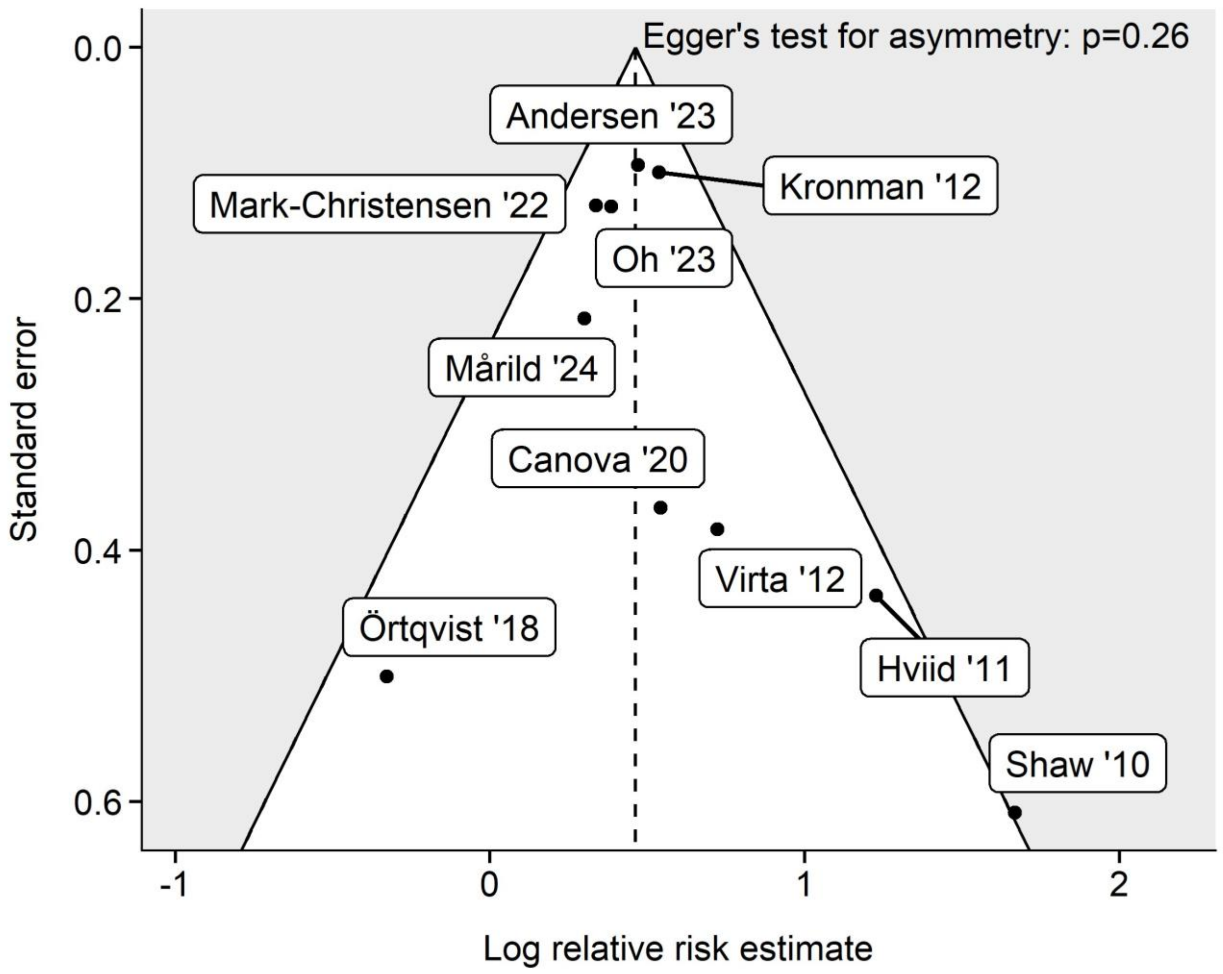


**Figure S2.** Funnel plot of childhood antibiotic exposure and risk of Crohn's disease. Each point represents a study-specific relative risk estimate (log HR, log OR, or log RR) plotted against its standard error. The dashed vertical line shows the pooled log RR. Diagonal lines depict the expected 95% range assuming no heterogeneity. Points falling within the grey area may indicate outliers, small-study effects, or between-study heterogeneity. HR = hazard ratio, OR = odds ratio, RR = risk ratio.

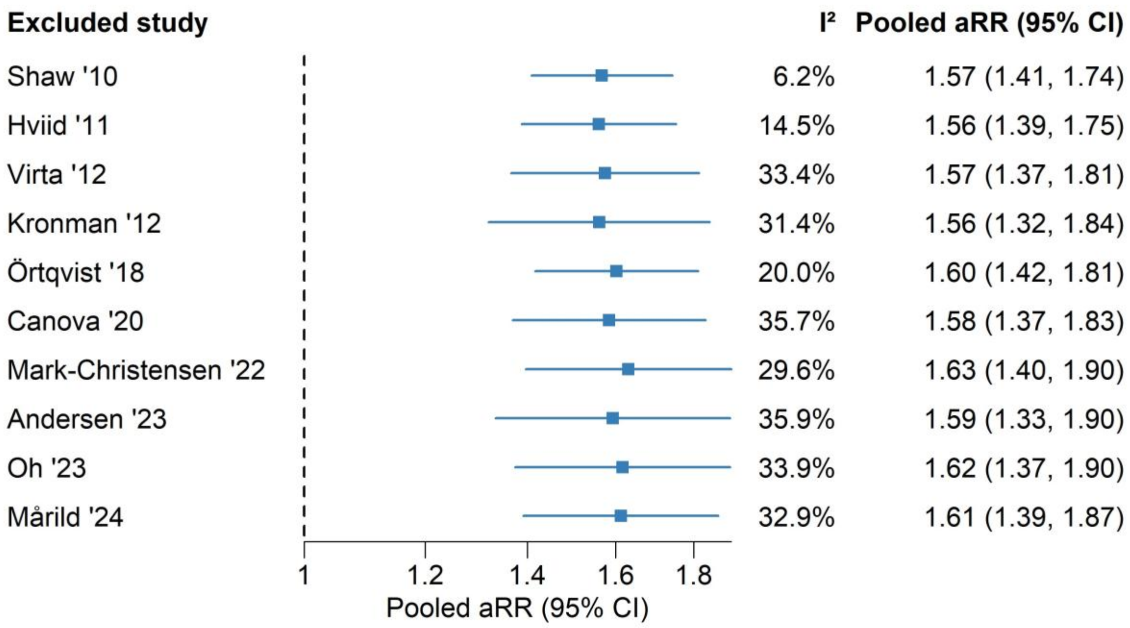


**Figure S3.** Leave-one-out sensitivity analysis of childhood antibiotic exposure and risk of Crohn's disease. Squares and horizontal lines represent the pooled adjusted risk ratio (aRR) and 95% confidence interval (CI) after excluding the indicated study. $I^2$ values represent between-study heterogeneity after exclusion of the respective study.

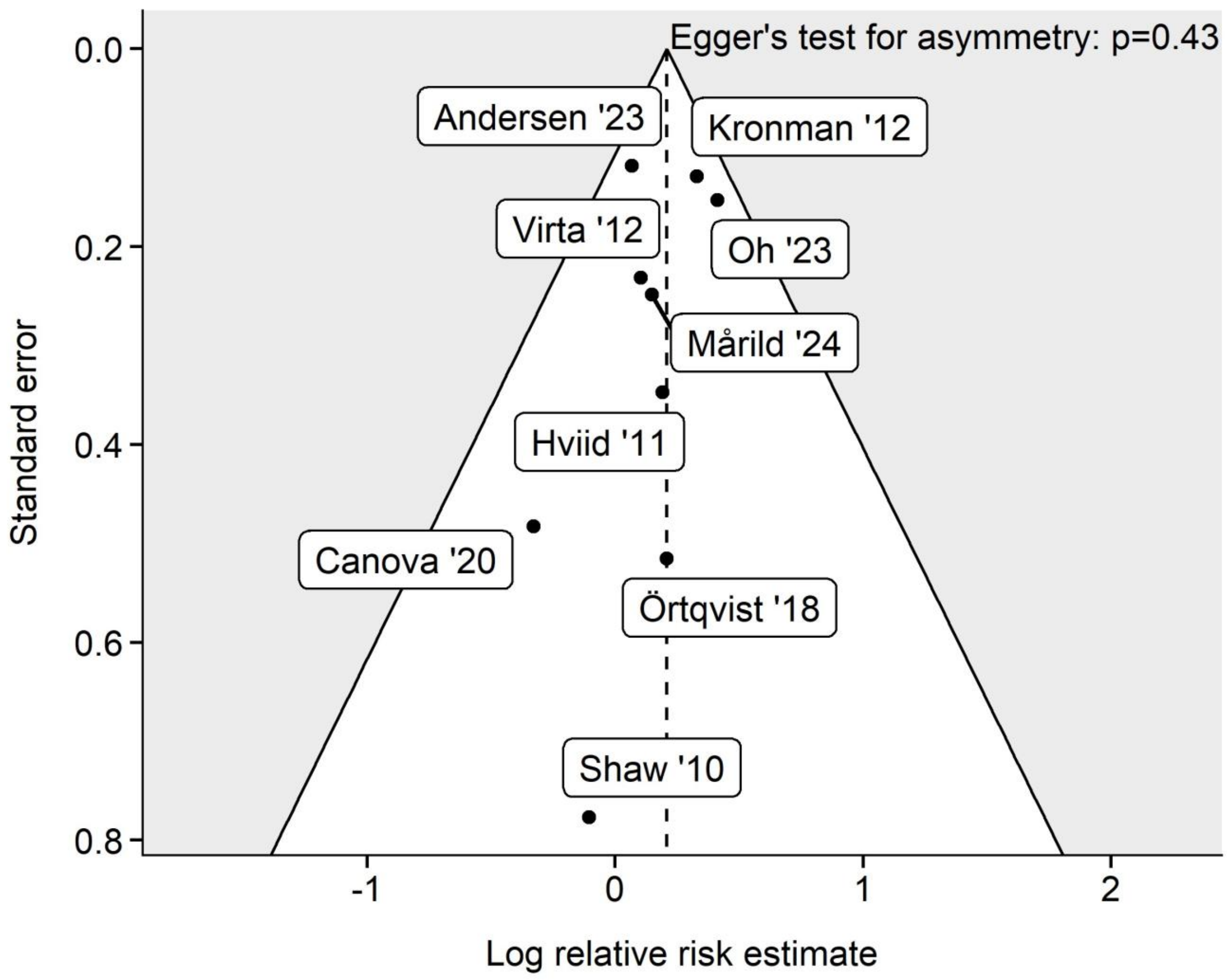


**Figure S4.** Funnel plot of childhood antibiotic exposure and risk of ulcerative colitis. Each point represents a study-specific relative risk estimate (log HR, log OR, or log RR) plotted against its standard error. The dashed vertical line shows the pooled log RR. Diagonal lines depict the expected 95% range assuming no heterogeneity. Points falling within the grey area may indicate outliers, small-study effects, or between-study heterogeneity. HR = hazard ratio, OR = odds ratio, RR = risk ratio.

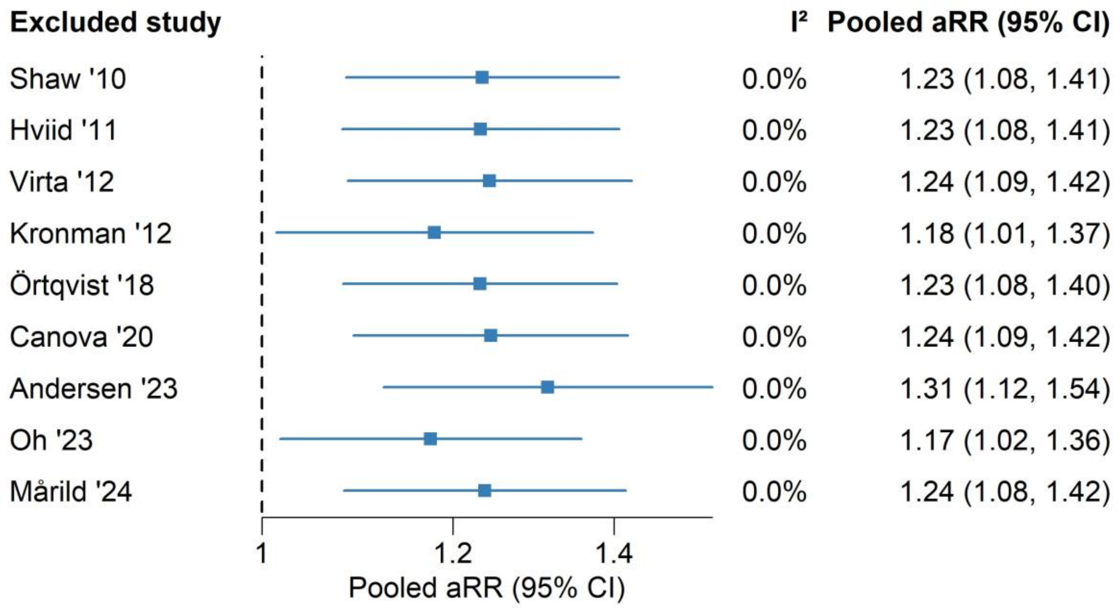


**Figure S5.** Leave-one-out sensitivity analysis of childhood antibiotic exposure and risk of ulcerative colitis. Squares and horizontal lines represent the pooled adjusted risk ratio (aRR) and 95% confidence interval (CI) after excluding the indicated study. $I^2$ values represent between-study heterogeneity after exclusion of the respective study.

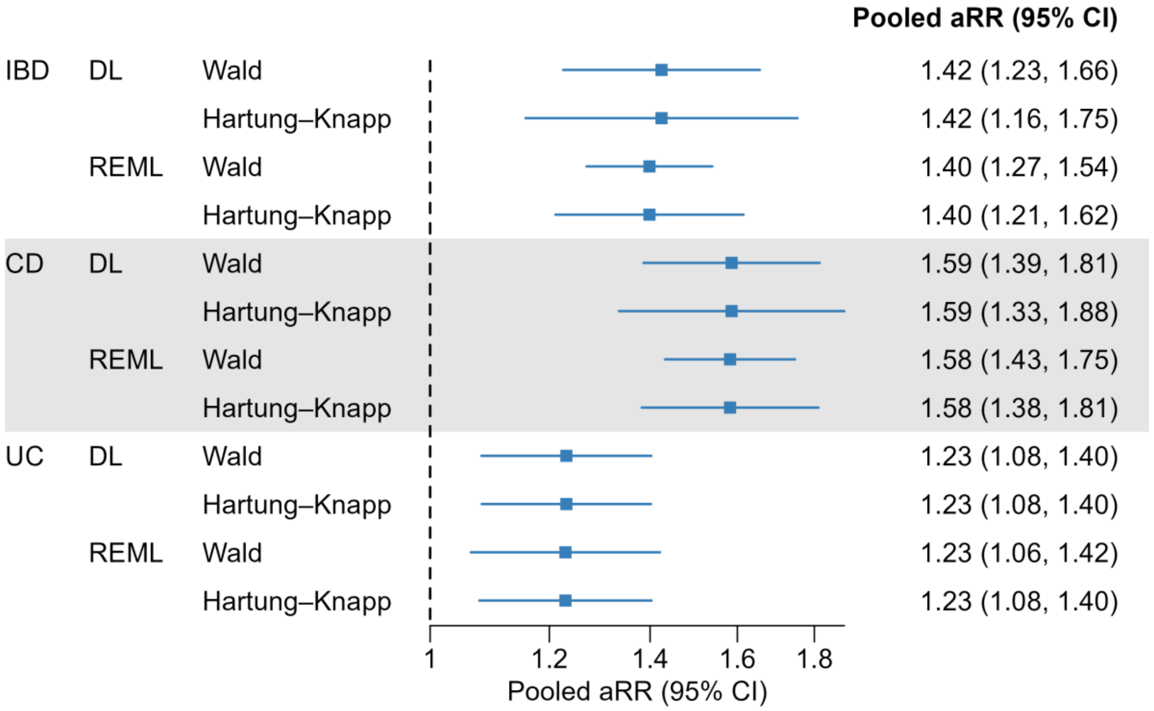


**Figure S6.** Sensitivity analyses of pooled adjusted risk ratios (aRRs) for inflammatory bowel disease (IBD), Crohn's disease (CD), and ulcerative colitis (UC) using alternative random-effects estimators and confidence interval methods. Pooled estimates were calculated using the DerSimonian–Laird (DL) and restricted maximum likelihood (REML) estimators with Wald-type and Hartung–Knapp confidence intervals. DL with Wald-type confidence intervals was used in the main analyses.

**References**


1. Shaw SY, Blanchard JF, Bernstein CN. Association between the use of antibiotics in the first year of life and pediatric inflammatory bowel disease. The American journal of gastroenterology. 2010;105(12):2687-92.
2. Hviid A, Svanström H, Frisch M. Antibiotic use and inflammatory bowel diseases in childhood. Gut. 2011;60(1):49-54.
3. Kronman MP, Zaoutis TE, Haynes K, Feng R, Coffin SE. Antibiotic exposure and IBD development among children: a population-based cohort study. Pediatrics. 2012;130(4):e794-e803.
4. Ortqvist AK, Lundholm C, Halfvarson J, Ludvigsson JF, Almqvist C. Fetal and early life antibiotics exposure and very early onset inflammatory bowel disease: a population-based study. Gut. 2018.
5. Canova C, Ludvigsson JF, Di Domenicantonio R, Zanier L, Barbiellini Amidei C, Zingone F. Perinatal and Antibiotic Exposures and the Risk of Developing Childhood-Onset Inflammatory Bowel Disease: A Nested Case-Control Study Based on a Population-Based Birth Cohort. Int J Environ Res Public Health. 2020;17(7).
6. Andersen S, Hestetun SV, Bernklev T, Perminow G, Stordal K. Fetal and Childhood Antibiotics and Risk of Pediatric Inflammatory Bowel Disease: A Population-Based Nationwide Register Study. J Pediatr Clin Pract. 2024;12:200096.
7. Oh SJ, Kim HJ, Lee CK, Big Data Research Group of the Korean Society of G. A dose-dependent increase in the risk of inflammatory bowel disease after exposure to broad-spectrum antibiotics: A national population study in Korea. Aliment Pharmacol Ther. 2023;58(2):191-206.
8. Marild K, Lerchova T, Ostensson M, Imberg H, Stordal K, Ludvigsson J. Childhood Infections, Antibiotics and Later Risk of Childhood and Early Adult-Onset Inflammatory Bowel Disease: Pooled Analysis of Two Scandinavian Birth Cohorts. Aliment Pharmacol Ther. 2025;61(2):323-34.
9. Virta L, Auvinen A, Helenius H, Huovinen P, Kolho K-L. Association of repeated exposure to antibiotics with the development of pediatric crohn's disease - A nationwide, register-based Finnish case-control study. Am J Epidemiol. 2012;175(8):775-84.
10. Mark-Christensen A, Lange A, Erichsen R, Frøslev T, Esen B, Sørensen HT, Kappelman MD. Childhood Exposure to Antibiotics and Risk for Crohn's Disease: A Nationwide Danish Birth Cohort Study. Inflamm Bowel Dis. 2022;28(3):415-22.
